# Recent Progress and Perspectives in the Electrokinetic Characterization of Polyelectrolyte Films

**DOI:** 10.3390/polym8010007

**Published:** 2015-12-31

**Authors:** Ralf Zimmermann, Carsten Werner, Jérôme F. L. Duval

**Affiliations:** 1 Leibniz Institute of Polymer Research Dresden, Max Bergmann Center of Biomaterials Dresden, Hohe Strasse 6, 01069 Dresden, Germany; werner@ipfdd.de; 2 Technische Universität Dresden, Center for Regenerative Therapies Dresden, Tatzberg 47, 01307 Dresden, Germany; 3 Laboratoire Interdisciplinaire des Environnements Continentaux (LIEC), CNRS UMR 7360, 15 avenue du Charmois, B.P. 40, F-54501 Vandoeuvre-lès-Nancy cedex, France; jerome.duval@univ-lorraine.fr

**Keywords:** electrokinetics, polyelectrolyte films, interfacial charge, streaming current, surface conductivity

## Abstract

The analysis of the charge, structure and molecular interactions of/within polymeric substrates defines an important analytical challenge in materials science. Accordingly, advanced electrokinetic methods and theories have been developed to investigate the charging mechanisms and structure of soft material coatings. In particular, there has been significant progress in the quantitative interpretation of streaming current and surface conductivity data of polymeric films from the application of recent theories developed for the electrohydrodynamics of diffuse soft planar interfaces. Here, we review the theory and experimental strategies to analyze the interrelations of the charge and structure of polyelectrolyte layers supported by planar carriers under electrokinetic conditions. To illustrate the options arising from these developments, we discuss experimental and simulation data for plasma-immobilized poly(acrylic acid) films and for a polyelectrolyte bilayer consisting of poly(ethylene imine) and poly(acrylic acid). Finally, we briefly outline potential future developments in the field of the electrokinetics of polyelectrolyte layers.

## 1. Introduction

The immobilization of polyelectrolytes is a versatile method for tailoring the surface properties of bulk materials. The spectrum of coatings ranges from non-ordered, adsorptively-bound polyelectrolytes over tethered polyelectrolyte brushes to polyelectrolyte multilayers formed by the alternate physisorption of polycationic and polyanionic polymers onto planar or colloidal supports [1,2]. Applications of polyelectrolyte-coated surfaces include, e.g., antimicrobial materials [3], stimuli-responsive surfaces [4], liquid displays [5], drug delivery [6], biosensing [7] and tissue-engineering scaffolds [8]. Capsules fabricated from the layer-by-layer adsorption of polyelectrolytes onto a particulate template prior to subsequent removal of the core are used as multifunctional carrier systems, e.g., for drug delivery [9]. Optimizing the formation, stability and performance of polyelectrolyte coatings necessarily includes the analysis of the interrelations between their charge, structure and interactions in a broad range of environmental conditions (pH, salt concentration, nature of ions present in the solution). Electrokinetic methods are well-established and valuable techniques to achieve such a level of understanding [10,11]. The interpretation of electrokinetic data requires theoretical formalisms that go beyond the classical concept of zeta potential [12], and instead, it necessitates the account of hydrodynamics within the soft polymer coatings under lateral flow conditions. Theories for electric double layer (EDL) and electrokinetics at soft (*i.e.*, ion- and water-permeable) surfaces were mainly developed in the last three decades [13,14,15,16,17,18,19,20,21,22,23,24,25,26,27,28]. Advanced theories are now available for the adequate physical interpretation of electrokinetic data collected at soft polymer/solution interfaces [21,22,23,24,25,26,27,28]. Applying such theories, it becomes possible to unambiguously unravel the electrostatic, hydrodynamic and structural properties of, e.g., micrometer-thick hydrogels [21,22,23], ionic-strength/pH-responsive thin polymer coatings [25], thermo-responsive thin films [24] or surface-supported brushes [28] carrying (or not) ionizable groups.

In this article, we review the fundamentals for the characterization of planar polyelectrolyte coatings by streaming current and surface conductivity measurements. We provide an overview of existing theory and of state-of-the-art experimental techniques used to analyze the charge and structure of polyelectrolyte systems. The impacts of the polymer charge, film permeability and segment density distribution on the measured streaming current are further illustrated and discussed on the basis of computational examples. To demonstrate the options arising from the recent theoretical developments, we discuss strategies and results reported for the investigation of plasma-immobilized poly(acrylic acid) films and for a polyelectrolyte bilayer consisting of poly(ethylene imine) and poly(acrylic acid). Finally, we briefly invoke a few potential future developments in the field.

## 2. Fundamental Principles and Theory

### 2.1. Streaming Current/Potential in Rectangular Microchannels

**Figure 1:**
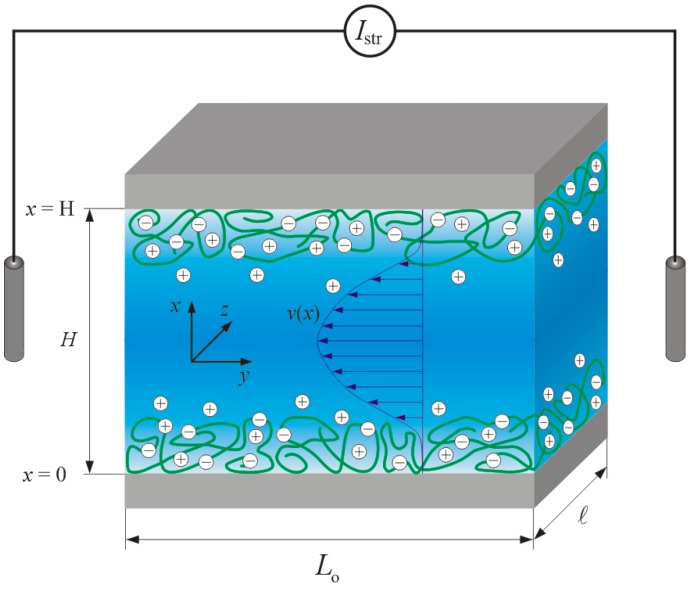
Schematic representation of a rectangular streaming channel for the characterization of soft polymer films supported by hard planar substrates from streaming current/potential measurements. The hydrodynamic flow field, *v*(*x*), developing in the channel under a lateral pressure gradient is indicated. The dimensions of the channel and polymer film are not shown to scale. For the sake of simplicity, charges located at the carrier surface are not represented. The scheme further details the nomenclature used in the theory reviewed in Section 2. Typical dimensions of the cell are: *L*_o_ = 20 mm, ℓ = 10 mm and *H* = 5 µm to 60 µm. Reprinted from [11]. Copyright 2013 with permission from Elsevier.

The ionization of functional groups distributed in adsorbed or covalently immobilized polyelectrolytes leads to the development of an interfacial excess charge. This charge is compensated by counter ions located in the adjacent electrolyte medium, and an electrical double layer is formed. Under the action of an applied lateral hydrodynamic flow field, the counter ions are displaced in the flow direction due to friction forces. In capillary systems, the convective ion transport originates a streaming current, $I_{\text{str}}$, that can be measured with an ampere meter via two non-polarizable (e.g., Ag/AgCl) electrodes positioned at the inlet and outlet side of the measuring cell (Figure 1). At high ohmic resistance of the external circuit, an electrical potential, the streaming potential, $U_{\text{str}}$, may be measured, provided that an electrometer is connected to the two electrodes. The magnitude of the streaming potential is governed by the steady-state balance between the streaming current and a back conduction current determined by the specific electrical conductivity of the electrolyte embedded in the capillary system and by the surface conductivity that arises from mobile counter ions at the material surface [11].

For the analysis of the interfacial charging and structure of soft polymer films, streaming current and streaming potential are performed under conditions of laminar flow across rectangular streaming channels that are formed by two identically-coated sample carriers of length *L*_o_ and width (Figure 1) [11]. To minimize the effects of side walls on the development of the hydrodynamic flow field within the channel, the separation distance between the two surfaces, *H*, is much smaller than the length and width of the surfaces (*H << L*_o_ and *H <<* ℓ). Typical dimensions of the streaming channel are *L*_o_ = 20 mm, ℓ = 10 mm and *H* = 30 µm [29,30]. To eliminate the contributions of the electrodes to streaming current/potential, measurements should be performed as a function of the applied pressure gradient along the surfaces of interest. Proceeding so, the contributions of the electrodes (due to asymmetry) can be eliminated upon evaluation of the ratio streaming current/potential over the applied pressure gradient. It is stressed that measured electrokinetic response varies linearly with the applied pressure, except for situations where the investigated interface is electroactive and (reversible) redox active species are present in the neighboring electrolyte solution [31,32].

Below, we review the fundamentals for the analysis of the streaming current and surface conductivity of soft (non-conducting) polymer films [23,24,25,26,27,28]. The theory was developed for steady-state electrostatic and hydrodynamic flow fields, and it is applicable for polymer films of sufficiently high water content [23,24,25,26,27,28]. The typical separation distance adopted between the two sample carriers excludes any overlap of the electric double layers formed between the soft films and the electrolyte solution embedded in the channel. Numerical and analytical procedures for solving the set of coupled equations determining the electrostatic potential and the hydrodynamic flow field distributions across the channel are described elsewhere [24].

### 2.2. Electrostatic Potential

The electrical potential across the substrate/polyelectrolyte (PE) layer/electrolyte solution interface, ψ(x), is a function of the local charge density at that position *x* with *x* referring to the dimension perpendicular to the interface (Figure 1). The charges of the PE-supporting substrate and those stemming from the ionizable groups carried by the PE layer are compensated by mobile counter ions distributed inside and outside the permeable layer structure. The electrolyte is considered to comprise *N* ions of valence *z_i_* with bulk concentrations *c_i_*, with the index *i* running from *i* = 1,…, *N*. The position-dependent electrostatic potential across a PE layer carrying *M* types of ionizable groups can be evaluated from solution of the non-linear Poisson–Boltzmann equation [26]:

(1) $$\frac{{\text{d}}^{2}y\left(X\right)}{\text{d}X^{2}}=-\frac{{\left(\kappa H\right)}^{2}}{\sum_{i=1}^{N}c_{i}{z_{i}}^{2}}\left\{\sum_{i=1}^{N}z_{i}c_{i}e^{-z_{i}y\left(X\right)}+\sum_{j=1}^{M}\frac{\rho_{j}/F}{1+10^{-\varepsilon_{j}\left(\text{p}K_{j}-\text{pH}\right)}e^{\varepsilon_{j}y\left(X\right)}}f\left(X\right)\right\},$$

where *y* is the dimensionless electrostatic potential (y=Fψ/RT), κ the reciprocal Debye length, *F* the Faraday constant, *R* the gas constant and *T* the temperature, and *X* corresponds to the dimensionless space variable *x*/*H*. The ionization of the *j*-th type of functional groups (*j* = 1,…, *M*) is defined here by their dissociation p*K_j_* value, and for the sake of conciseness, we consider that charged groups carry a unique charge ±*e*. The parameter $\varepsilon_{j}$ involved in Equation (1) is −1 for the ionization of functional groups according to deprotonation reactions (*i.e.*, of the type $-{\text{RH}}_{j}⇄-{{\text{R}}_{j}}^{-}+{\text{H}}^{+}$), and $\varepsilon_{j}$ is +1 for the ionization of groups due to protonation reactions (*i.e.*, $-{{\text{RH}}_{j}}^{+}⇄-{\text{R}}_{j}{+\text{H}}^{+}$) [26]. The function f(x) in Equation (1) represents the polymer segment density distribution across the soft interfacial layer. Analytical expressions or numerical values for f(x) may be derived from polymer theory [28] or determined experimentally [33,34,35]. Measurements performed with spatially-resolved techniques, such as ellipsometry or neutron/X-ray reflectivity [33,34,35], showed that the polymer segment density profile can be appropriately represented by the function:

(2) $$f\left(x\right)=\frac{\omega }{2}\left(1-\tanh\left(\frac{x-d}{\alpha }\right)\right),$$

where *α* is the characteristic decay length of the segment density across the soft layer [24] and *d* is the nominal surface layer thickness reached in the limit α→0. The scalar quantity *ω* ensures that the total amount of polymer segments within the soft film remains constant when the film structure/thickness vary as a result of shrinking/swelling processes driven by changes in solution pH, salt concentration or temperature [24] and accompanied by modulations of *α*. In the limiting case of a homogeneous segment distribution, *i.e.*, α/d→0, Equation (2) corresponds to a step function-like profile [24], and the film protrudes from the supporting solid substrate up to a distance *d*. Under such conditions, the charge source term, $\left|\rho_{j=1,...,M}\right|/F$, in Equation (1) corresponds to the total concentration of ionizable groups of type *j* within the layer. The boundary condition at X=0 associated with the non-linear differential Equation (1) can be expressed in terms of the surface potential (or zeta potential ζ) or surface charge density of the *hard* (impermeable) carrier supporting the film [25]. If the charge of the substrate does not significantly contribute to the streaming current and surface conductivity, zero electric field condition may be imposed at X=0 [25]. For practically relevant systems where the separation distance between the channel walls is much larger than the extension of the PE layers and the electric Debye layer thickness, the overlap of the electrical double layers is excluded. As a result of electroneutrality far from the interface, the second boundary condition required for solving Equation (1) is simply *y* (*X* → 1/2) = 0.

### 2.3. Hydrodynamic Flow Field

The hydrodynamic flow profile *ν*(*x*) within the thin electrokinetic cell can be evaluated on the basis of the Brinkman equation [36]. For rectangular channels with a separation distance *H* much smaller than the length *L*_o_ and width ℓ of the channel, the flow field is oriented parallel to the sample surfaces and symmetric with respect to the *y*-*z*-plane at x=H/2. Provided that sample carriers are coated with identical polymer films, it is therefore sufficient to consider the hydrodynamic problem from X=0 to X=1/2. Under these conditions the Brinkman equation may be written in the form [25]:

(3) $$\frac{{\text{d}}^{2}V\left(X\right)}{\text{d}X^{2}}-{\left(\lambda_{\text{o}}H\right)}^{2}f\left(X\right)V\left(X\right)=-1,$$

where the velocity field *v* is normalized according to $V\left(X\right)=v\left(X\right)/v_{\text{o}}$ with $v_{\text{o}}=\Delta PH^{2}/\left(\eta L_{o}\right)$, where *η* is the dynamic viscosity of the electrolyte. In Equation (3), $\lambda_{\text{o}}$ corresponds to the hydrodynamic softness of the polymer film. Its reciprocal value, $1/\lambda_{\text{o}}$, is the Brinkman length that reflects the extension of the hydrodynamic flow field into the soft film in case of homogeneous segment density distribution, *i.e.* α/d→0 and ω→1. Equation (3) is applicable for the analysis of soft polymer films with sufficiently high water content, a situation frequently met in practice. The friction exerted by the polymer segments located at the position *x* on the fluid flow is then determined by the term ${\left(\lambda_{\text{o}}H\right)}^{2}f\left(X\right)$. For systems with higher segment density, higher order density-dependent friction terms have to be taken into account [28]. The boundaries necessary to solve the Brinkman Equation (3) are given by the no-slip condition at the supporting hard carrier surface (*X* = 0) and by the symmetry of the flow field at the position *X* = 1/2 [25].

### 2.4. Streaming Current and Streaming Potential

The streaming current *I*_str_ can be calculated from the spatial integral of the product between the charge density distribution of counter ions (first term on the right-hand side of Equation (1)) and the hydrodynamic flow field, *V*(*X*) (Equation (3)), according to [24,25]:

(4) $$\frac{I_{\text{str}}}{\Delta P}=\frac{2\ell FH^{3}}{\eta L_{\text{o}}}\int_{0}^{1/2}\sum_{i=1}^{N}z_{i}c_{i}e^{-z_{i}y\left(X\right)}V\left(X\right)\;\text{d}X.$$

Analytical expressions for $I_{\text{str}}/\Delta P$ may be derived under conditions in line with the application of the Debye–Hückel approximation (valid for sufficiently low potentials *y*) and for films with a homogeneous segment density distribution [24]. For hard surfaces, the solution of Equation (4) leads to the famous Smoluchowski equation that relates the streaming current to the electrokinetic or zeta potential [11]. Equation (4) can be converted into an expression for the streaming potential $U_{\text{str}}$ via Ohm’s law, taking into account the terms for the specific electrical conductivity of the electrolyte and for the surface conductivity [11].

### 2.5. Surface Conductivity

The accumulation of mobile counter ions inside and outside a charged soft surface layer leads to elevated electrical conductivity in the substrate/PE/electrolyte interfacial region that is designated as (excess) surface conductivity, $K^{\sigma }$. This excess surface conductivity originates from the (lateral) migrative transport of ions located in the interfacial electric double layer due to the tangential field $U_{\text{str}}/L$ (term $K_{m}^{\sigma }$):

(5) $$K_{\text{m}}^{\sigma }=\frac{HF^{2}}{RT}\int_{0}^{1/2}\sum_{i=1}^{N}\left|z_{i}\right|D_{i}c_{i}\beta_{i}\left(X\right)\left[e^{-z_{i}y\left(X\right)}-1\right]\text{d}X,$$

and from the electro-osmotic charge transport due to the action of the field $U_{\text{str}}/L_{\text{o}}$ on mobile ions within and outside the soft surface layer (term $E_{eo}^{\sigma }$):

(6) $$K_{\text{eo}}^{\sigma }=\frac{H\varepsilon_{\text{o}}\varepsilon_{\text{r}}RT{\left(\kappa H\right)}^{2}}{\eta }\int_{0}^{1/2}V_{\text{eo}}\left(X\right)\sum_{i=1}^{N}z_{i}c_{i}e^{-z_{i}y\left(X\right)}\text{d}X,$$

where *D_i_* is the diffusion coefficient of ion i in the bulk electrolyte solution, $\beta_{i}\left(X\right)$ the ratio between the mobilities of ion *i* at position *X* and in the bulk electrolyte solution, and $\varepsilon_{\text{o}}\varepsilon_{\text{r}}$ is the dielectric permittivity of the medium [25]. For soft films with sufficiently low polymer density it is legitimate to assume $\beta_{i}\left(X\right)∼1$. The electro-osmotic flow field, *V*_eo_(*X*), can be calculated on the basis of the Brinkman equation (3) after substituting the right hand side by the relevant charge source term and *V(X)* by *V*_eo_(*X*) [25,26]. The overall surface conductivity, $K^{\sigma }$, is then given by the sum $K_{\text{m}}^{\sigma }+K_{\text{eo}}^{\sigma }$.

In case of rectangular streaming channels (Figure 1), the surface conductivity can be determined from streaming current and streaming potential measurements at a single or several channel heights according to the following equation:

(7) $$\frac{I_{\text{str}}/\Delta P}{U_{\text{str}}/\Delta P}\frac{L_{\text{o}}}{2\ell }=\frac{H}{2}K^{\text{B}}+{Κ}^{\sigma },$$

where $K^{\text{B}}$ is the specific electrical conductivity of the bulk electrolyte [29]. The ratio of the pressure-normalized streaming current ($I_{\text{str}}/\Delta P$) and streaming potential ($U_{\text{str}}/\Delta P$) represents the channel conductance. This quantity can be alternatively determined under conditions of zero flow from the measurement of the electrical current when applying externally an electrical potential difference along the microchannel [23]. If the channel conductance is evaluated at several channel heights, $K^{\sigma }$ then simply corresponds to the intercept in the (linear) plot channel conductance *vs.* channel height [29] and $K^{\text{B}}$ directly follows from the slope. The comparison between so-determined $K^{\text{B}}$ and values estimated from the concentrations and known molar conductivities of the ions present in solution can be helpful for verification purposes.

### 2.6. Surface Conductivity and Donnan Potential

Polyelectrolyte layers can be treated as a 3D meshwork where ionizable groups are immobilized. The ionization of these groups is related to an enhanced concentration of (counter) ions within the layer, which in turn causes an electrical potential difference between the PE layer phase and the electrolyte. For sufficiently thick PE layers, *i.e.*, whose thickness is much larger than the screening Debye length 1/κ, with a homogeneous segment distribution (*i.e.*, α/d→0), the potential difference can be viewed as the analogue of the Donnan potential difference across a semipermeable membrane [37]. Dukhin *et al*. provided analytical expressions that relate a given concentration of ionizable groups in the PE layer (denoted here as $\left|\rho_{0}\right|/F$) to the dimensionless Donnan potential $y_{\text{D}}$, across the PE layer/electrolyte solution interface [38]. For PE layers with anionic groups (negative $y_{\text{D}}$), the corresponding equations can be written in the form:

(8) $${\text{e}}^{-y_{\text{D}}}=\sqrt{\frac{\left|\rho_{0}\right|}{cF}10^{pH-pK}+1}\;\mathrm{for}\;\left|y_{\text{D}}\right|<2$$

(9) $${\text{e}}^{-y_{\text{D}}}=\frac{10^{pH-pK}}{2}\left(\sqrt{1+4\frac{\left|\rho_{0}\right|}{cF}10^{pK-pH}}-1\right)\;\mathrm{for}\;\left|y_{\text{D}}\right|>2$$

where *c* is the total ion concentration in the bulk electrolyte (ions of the neutral background electrolyte and ions resulting from the variation of the solution pH have to be taken into account) [38]. Under the conditions specified above, the concentration of ionizable groups, $\left|\rho_{0}\right|/F$, can be quantified by measuring the surface conductivity and layer thickness under conditions of complete ionization. Equations (8) and (9) then allow for the evaluation of pH and salt concentration effects on the magnitude of the Donnan potential.

## 3. Electrokinetic Analysis of Polyelectrolyte Layers

### 3.1. Illustrative Computational Examples

**Figure 2:**
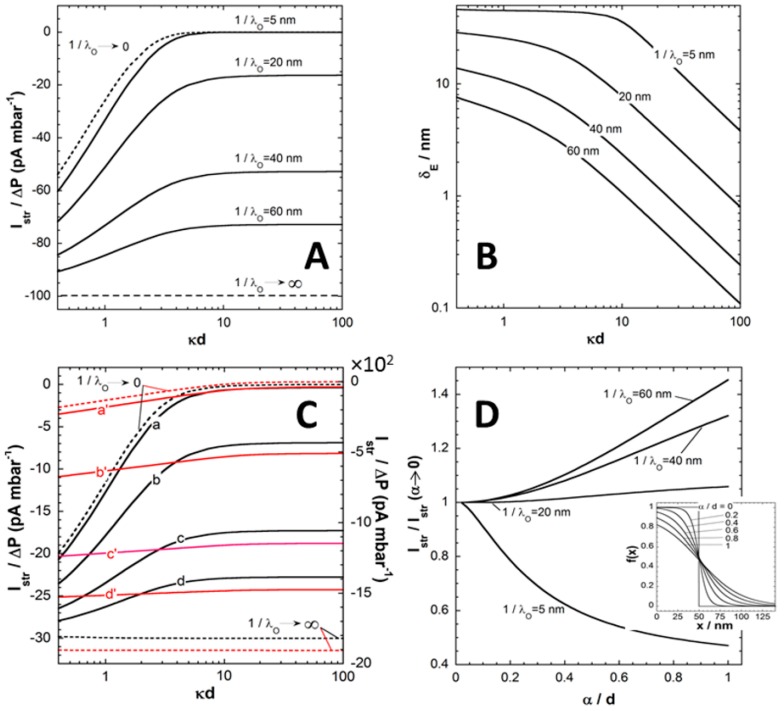
(**A**) Ratio between streaming current ($I_{\text{str}}$) and applied pressure (ΔP ) *versus* ionic strength evaluated from theory for different values of the hydrodynamic penetration length $1/\lambda_{\text{O}}$ (indicated). (**B**) Electrokinetic layer thickness $\delta_{\text{E}}$ estimated from theory under the conditions of panel **A** at various $1/\lambda_{\text{O}}$ (indicated). Model parameters in (**A**) and (**B**): ζ=−90 mV, z=1 (monovalent electrolyte), T = 22 °C, d=50 nm, H=30 μm, ℓ=10 mm, $L_{\text{O}}=20$ mm, $\varepsilon_{\text{r}}=79.5$, η=0.954 mPa s^-1^, α→0 (homogeneous segment density distribution in the film), $\rho_{\text{O}}=0$ (uncharged film). (**C**) $I_{\text{str}}/\Delta P$ *versus* ionic strength evaluated from theory for $\left|\rho_{\text{O}}/F\right|=0.1$ mM (black lines, left *y*-axis) and $\left|\rho_{\text{O}}/F\right|=10$ mM (red lines, right *y*-axis) at (a, a’) $1/\lambda_{\text{O}}=5$ nm, (b, b’) $1/\lambda_{\text{O}}=20$ nm, (c, c’) $1/\lambda_{\text{O}}=40$ nm and (d, d’) $1/\lambda_{\text{O}}=60$ nm. The limits $1/\lambda_{\text{O}}\to 0$ and $1/\lambda_{\text{O}}\to \infty$ are explicitly indicated. Other model parameters: as in (**A**)-(**B**) except ζ=−10 mV (**D**) Ratio $I_{\text{str}}/I_{\text{str}}\left(\alpha \to 0\right)$ as a function of α/d at various $1/\lambda_{\text{O}}$ (indicated). Other model parameters: as in (**A**) and (**B**) except that ζ=−10 mV, $\left|\rho_{\text{O}}/F\right|=10$ mM and solution ionic strength = 1 mM. In the inset of panel (**D**), representation of the segment density distribution *f*(*x*) for various values of α/d (indicated) and d=50 nm. Adapted from [24]. Copyright 2009 with permission from American Chemical Society.

In this section, we briefly discuss the basic processes governing the dependence of the pressure-normalized streaming current, $I_{\text{str}}/\Delta P$, on electrolyte concentration, flow penetration length scale $1/\lambda_{\text{o}}$ (Figure 2A,B), charge density $\left|\rho_{0}\right|$ in (anionic) polyelectrolyte film (Figure 2C) and segment density distribution therein (Figure 2D). For the sake of demonstration, we consider a monovalent electrolyte of bulk concentration *c* and films with a given (negative) density of charges (*i.e.*, no variation of the film charge with the pH).

In order to first highlight the hydrodynamic contribution of the polyelectrolyte film (thickness *d*) to $I_{\text{str}}/\Delta P$, Figure 2A refers to the situation of an uncharged film ($\left|\rho_{0}\right|=0$) supported by a hard carrier with (negative) surface potential, the latter identifying with the carrier zeta-potential ζ that can be measured in the absence of the film [24]. The ratio $I_{\text{str}}/\Delta P$ for this interfacial system is reported as a function of *c* expressed here in terms of the dimensionless quantity κd that compares the Debye layer thickness $\kappa^{-1}={\left[2F^{2}c/\left(\varepsilon_{\text{o}}\varepsilon_{\text{r}}RT\right)\right]}^{-1/2}$ with the film thickness *d*. At fixed κd, Figure 2A illustrates that $I_{\text{str}}/\Delta P$ increases in magnitude with increasing $1/\lambda_{\text{o}}$. Upon increase of the penetration of the pressure-driven flow in the film, which is related to a decrease of the film hydrodynamic drag, the counter ions located in the vicinity of the charged supporting substrate become accessible to the flow and $I_{\text{str}}/\Delta P$ increases accordingly. The amount of electrokinetically active ions thus increases with increasing $1/\lambda_{\text{o}}$, and so does $I_{\text{str}}/\Delta P$. As expected, at fixed $1/\lambda_{\text{o}}$ $\left|I_{\text{str}}/\Delta P\right|$ decreases with increasing κd because of the screening of the carrier surface charge by ions present in the medium. Doing so, $I_{\text{str}}/\Delta P$ reaches at sufficiently large κd an asymptotic plateau, the value of which is determined by $1/\lambda_{\text{o}}$. In this limit where κd>>1, the electric double layer significantly recedes within the film coating and $I_{\text{str}}/\Delta P$ becomes essentially determined by details of flow velocity and electrostatic potential distributions in the direct neighborhood of the carrier surface. Under such conditions, it can be shown that the streaming current reduces to [24]:

(10) $$I_{\text{str}}=\frac{\beta \tilde{\zeta }}{\left[2{\left(\kappa H\right)}^{2}\right]}\left\{\frac{1}{\cosh\left(\lambda_{\text{o}}d\right)}+\frac{2}{\lambda_{\text{o}}H}\tanh\left(\lambda_{\text{o}}d\right)\right\},$$

where $\beta =4\ell Fc\Delta PH^{3}/\left(\eta L_{\text{o}}\right)$ and $\tilde{\zeta }=\zeta F/RT$is the dimensionless zeta-potential of the substrate. In the extreme $1/\lambda_{\text{o}}\to \infty$, the situation is that of a free-draining film fully permeable to flow. From a hydrodynamic point of view, the (uncharged) film is thus immaterial and its presence does not affect the electrokinetic signature of the rigid surface. The streaming current then simplifies into the well-known Smoluchowski formulation $I_{\text{str}}=\beta \tilde{\zeta }/\left[2{\left(\kappa H\right)}^{2}\right]$. In the other limit where $1/\lambda_{\text{o}}\to 0$, the film is *stricto sensu* impermeable. The streaming current can then be written as a function of the electrostatic potential at the outer film surface (x=d in Figure 1) *via* the modified Smoluchowski equation $I_{\text{str}}=\beta \tilde{\zeta }e^{-\kappa d}/\left[2{\left(\kappa H\right)}^{2}\right]$ that holds at sufficiently low $\tilde{\zeta }$ where linearization of the Poisson-Boltzmann equation is legitimate. Figure 2A makes it clear that the screening of the carrier surface charge by the film is defined by the interplay between 1/λ_o_ and *d* (hydrodynamic screening), and by the respective magnitudes of 1/κ and *d* (electrostatic screening).

Another way to formulate these two types of processes is to define the so-called electrokinetic thickness of the film coating, denoted as *δ*_E_ and expressed *via* the following relationship

(11) $$\tanh\left(F\bar{\zeta }/4RT\right)=\tanh\left(F\zeta /4RT\right)\exp\left(-\kappa \delta_{\text{E}}\right),$$

The effective electrokinetic potential $\bar{\zeta }$ involved in Equation (11) is obtained from the Smoluchowski-based conversion of the streaming current *rigorously* evaluated in the presence of the film layer, *i.e.* $\bar{\zeta }=2RT{\left(\kappa H\right)}^{2}I_{\text{str}}/\left(F\beta \right)$. The presence of the uncharged film leads to a partial or complete suppression of the flow in the vicinity of the charged rigid surface so that $\left|\bar{\zeta }\right|<\left|\zeta \right|$. In this picture, $\bar{\zeta }$ is thus basically determined from the outer shift of a virtual “plane of shear” for which the decrease in zeta potential is a measure, and this shift is identified to $\delta_{\text{E}}$. Examination of measured electrokinetic data on the basis of Equation (11) only, should be considered as phenomenological rather than based on physical arguments. Instead, the formulation given by Equation (11) necessarily implies that $\delta_{\text{E}}$ reflects the details of the electrostatic and hydrodynamic flow field profiles within the soft surface layer. Figure 2B collects the dependence of $\delta_{\text{E}}$ on κd under the conditions of Figure 2A. In line with the preceding arguments, at fixed $1/\lambda_{\text{o}}$ $\delta_{\text{E}}$ decreases with increasing κd because the electrokinetically active ions (*i.e.* probed by the tangential flow) becomes gradually confined to the close vicinity of the carrier surface, and the more so the larger $1/\lambda_{\text{o}}$ is. It is observed that at large κd, $\delta_{\text{E}}$ decreases linearly with κ (at fixed *d*) in double logarithmic representation. This trend is confirmed by the analytical expression that may be developed for $\delta_{\text{E}}$ at κd>>1 and low $\bar{\zeta }$ [24]:

(12) $$\delta_{\text{E}}=-\frac{1}{\kappa }\ln\left\{\frac{1}{\cosh\left(\lambda_{\text{o}}d\right)}+\frac{2}{\lambda_{\text{o}}H}\tanh\left(\lambda_{\text{o}}d\right)\right\},$$

In the limit κd<<1 where film thickness is small compared to the Debye layer extension from the rigid carrier surface, $\delta_{\text{E}}$ reaches a plateau whose value is solely determined by the hydrodynamic film permeability (Figure 2B). In this limit, $\delta_{\text{E}}$ identifies with the so-called hydrodynamic thickness $\delta_{\text{H}}$ of the film that further reduces to the physical film thickness *d* for situations where film resistance to flow is infinite (*i.e.* $1/\lambda_{\text{o}}$→0).

Figure 2C shows the evolution of $I_{\text{str}}/\Delta P$ on κd for cases where both supporting carrier and polyelectrolyte film are charged. Data are reported for various values of $1/\lambda_{\text{o}}$ with $\left|\rho_{0}\right|/F=0.1$ mM (poorly charged film, black lines) and $\left|\rho_{0}\right|/F=10$mM (red lines). At fixed $\left|\rho_{0}\right|/F$, the dependence of $I_{\text{str}}/\Delta P$ on κd and $1/\lambda_{\text{o}}$ is qualitatively similar to that discussed in Figure 2A. Quantitatively, the introduction of structural charges in the polyelectrolyte films goes in pair with an enhancement of the local electrostatic potential under given salt concentration conditions, and thus with an increase in the amount of counterions accumulated therein. As a result, the streaming current increases in magnitude and the larger $1/\lambda_{\text{o}}$ is, the most pronounced is this increase in $\left|I_{\text{str}}/\Delta P\right|$. The dependence of $I_{\text{str}}/\Delta P$ on κd becomes weaker as the density of charges carried by the film increases. This is due to a reduction of the electrostatic screening of the carrier surface charge as the electric double layer composition becomes increasingly governed by the electrostatic properties of the polyelectrolyte layer. Similarly to the situation analyzed in Figure 2A, $I_{\text{str}}/\Delta P$ reaches an asymptotic plateau at κd>>1 and the value of this plateau is not only a function of $\tilde{\zeta }$ and $1/\lambda_{\text{o}}$ (see Equation (10)) but also involves $\rho_{0}$ as detailed in [24]. Finally, the limits $1/\lambda_{\text{o}}\to 0$ and $1/\lambda_{\text{o}}\to \infty$ corresponding to impermeable and free-draining films, respectively, can be rationalized *via* a Smoluchowski-like equation of the form $I_{\text{str}}=\beta {\tilde{\psi }}_{\text{o}}/\left[2{\left(\kappa H\right)}^{2}\right]$ where the dimensionless potential ${\tilde{\psi }}_{\text{o}}=F\psi_{\text{o}}/RT$ is evaluated at x=d (case $1/\lambda_{\text{o}}\to 0$) or x=0 (case $1/\lambda_{\text{o}}\to \infty$). In both limits, it is stressed that ${\tilde{\psi }}_{\text{o}}$ is a function of both the surface carrier potential ζ and the film charge density$\rho_{0}$. As an illustration, in the limit of low potentials, ${\tilde{\psi }}_{\text{o}}$ is defined by ${\tilde{\psi }}_{\text{o}}=\left[\tilde{\zeta }+\frac{\rho_{0}}{2cF}\left(\cosh\left(\kappa d\right)-1\right)\right]{\text{e}}^{-\kappa d}$at $1/\lambda_{\text{o}}\to 0$ and ${\tilde{\psi }}_{\text{o}}=\tilde{\zeta }+F\rho_{0}d^{2}/\left(2\varepsilon_{\text{o}}\varepsilon_{\text{r}}RT\right)$at $1/\lambda_{\text{o}}\to \infty$, expressions that are derived from the relevant analytical formulations reported in [24]. In addition the former expression further reduces to ${\tilde{\psi }}_{\text{o}}=y_{\text{D}}/2$ for κd>>1 where $y_{\text{D}}/2$ corresponds to the (dimensionless) potential at the film surface (x=d) with $y_{\text{D}}=\rho_{0}/2cF$ the dimensionless Donnan potential in the Debye-Hückel approximation. 

Finally, we report in Figure 2D the impact of the interfacial film diffuseness α (see Equation (2), and insert in Figure 2D) on streaming current $I_{\text{str}}$ for various $1/\lambda_{\text{o}}$ values at fixed (monovalent) electrolyte concentration (c=1 mM). It is stressed that variations in α lead to modifications of the segment density profile f(x) as given in Figure 2D with the condition of constant total amount of polymer segments in the film (condition ensured *via* the scalar ω involved in Equation (2)). Results are given in the form $I_{\text{str}}\left(\alpha \right)/I_{\text{str}}\left(\alpha \to 0\right)$ *versus* α/d in order to reveal how $I_{\text{str}}$ evaluated under conditions where segment density distribution is diffuse, compares with that obtained for homogeneous polyelectrolyte film. At low to moderate $1/\lambda_{\text{o}}$, the ratio $I_{\text{str}}\left(\alpha \right)/I_{\text{str}}\left(\alpha \to 0\right)$ decreases with increasing α/d. This effect results from the friction exerted by the segments protruding from the film bulk phase on the flow in the region where flow velocity field is most significant. The reduction in streaming current thus results from an increased hydrodynamic drag of the outer film region and therewith from a marked immobilization of otherwise electrokinetically active ions.

On the opposite, at large $1/\lambda_{\text{o}}$ (typically $1/\lambda_{\text{o}}∼d$ or $1/\lambda_{\text{o}}>>d$), the ratio $I_{\text{str}}\left(\alpha \right)/I_{\text{str}}\left(\alpha \to 0\right)$ increases with increasing α/d. Indeed, extending the thickness of the diffuse interface also leads to an increase of the magnitude of the local electrostatic potential in the region x>d (see insert). For large $1/\lambda_{\text{o}}$, this electrostatic feature overwhelms the hydrodynamic modulations of the flow velocity associated to changes in segment density distribution and, consequently, it results in an increase of $I_{\text{str}}\left(\alpha \right)/I_{\text{str}}\left(\alpha \to 0\right)$ with increasing α/d. For values of $1/\lambda_{\text{o}}$ intermediate between the two aforementioned extremes, the balance between effects of α/d on interfacial hydrodynamics and electrostatics determines the magnitude of the ratio $I_{\text{str}}\left(\alpha \right)/I_{\text{str}}\left(\alpha \to 0\right)$ [24]. In situations of practical interest where $1/\lambda_{\text{o}}<d$, heterogeneity in the outer distribution of polymer segments density primarily causes a reduction in $\left|I_{\text{str}}\right|$ due to hydrodynamic screening, as further discussed below for poly(acrylic acid) thin films.

### 3.2. Charging and Structure of Poly(Acrylic Acid) Thin Films

The interfacial structure of PE films is governed by the level of ionization of their functional groups and the related increase in osmotic pressure. In this section, we review a strategy for the analysis of the charging and structure of surface-immobilized PE layers on the basis of streaming current, surface conductivity and swelling data measured at covalently-immobilized poly(acrylic acid) (PAA) films [26]. The PAA was attached to hard surfaces using a low-pressure plasma treatment [26].

**Figure 3:**
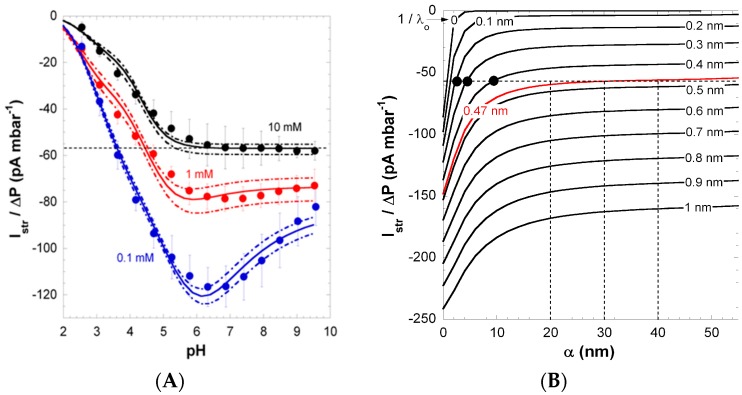
(**A**) Pressure-normalized streaming current, $I_{\text{str}}/\Delta P$, for PAA films as a function of pH in 0.1, 1 and 10 mM KCl solutions (indicated). Experimental data are represented by symbols. Solid and dotted lines were calculated on the basis of the theory by adjustment of the pH- and ionic-strength dependence of the interphasial diffuseness *α* that best brackets experimental values (dotted lines). (**B**) Dependence of $I_{\text{str}}/\Delta P$ on *α* and $1/\lambda_{\text{o}}$ at 10 mM KCl concentration under condition where full dissociation of the film charges is reached (pH > 6). For each $1/\lambda_{\text{o}}$ value tested, the value adopted for the charge density $\rho_{\text{o}}/F$ in the film is that which fits the surface conductivity $K^{\sigma }$ at large pH (see Figure 2). The unambiguous evaluation of $1/\lambda_{\text{o}}$ is done upon analysis of the intersection points between $I_{\text{str}}/\Delta P$ *vs. α* curves with the $I_{\text{str}}/\Delta P$ value measured at large pH in 10 mM KCl (dotted horizontal line in panel **A** and **B**), with as result $1/\lambda_{\text{o}}$ = 0.47 nm. The corresponding range of *α* consistent with $I_{\text{str}}/\Delta P$ at large pH in 10 mM KCl is then straightforwardly determined (see vertical dotted lines). The full black circles correspond to conditions that do not allow recovery of the $I_{\text{str}}/\Delta P$ *vs.* pH curve displayed in panel **A** (which was verified from theoretical simulations). Reprinted from [26]. Copyright 2011 with permission from Elsevier.

Figure 3 shows the pH-dependence of streaming current at KCl solution concentrations in the range 0.1 to 10 mM. According to the polymer structure, the charging of PAA films is dominated by the ionization of carboxyl groups in the repeat unit (please note that unsymmetrical water ion adsorption [39] can contribute to the overall film charge at low pH). In line with the highly anionic nature of PAA, the isoelectric point (IEP) of the films was found below pH 2. In 0.1 mM KCl solution, the streaming current exhibits a pronounced non-monotonous dependence on pH with the presence of a maximum at pH ~6 (Figure 3), while data collected at lower salt concentrations conform to commonly-reported trends (see below). The maximum in streaming current gradually vanishes with increasing KCl solution concentration. To decipher the reasons for this unconventional electrokinetic fingerprint, surface conductivity and film swelling were systematically measured under the conditions adopted for the electrokinetic experiments [26].

**Figure 4:**
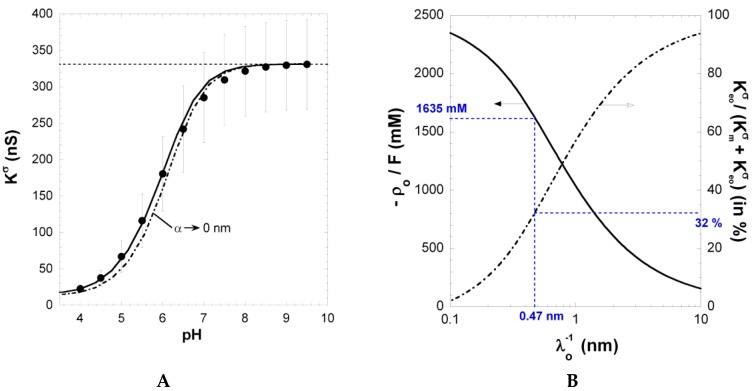
(**A**) Surface conductivity $K^{\sigma }$ *vs.* pH for PAA film in 1 mM KCl solution (symbols). The solid line represents the reconstruction of the data by theory. Parameters: $\rho_{\text{o}}/F=$ -1635 mM, $1/\lambda_{\text{o}}=$ 0.47 nm, $\text{p}K_{1}=$ 3.3, and *α* = 27 nm (solid line) as well as α→0 (dotted line). The plateau value reached by $K^{\sigma }$ at large pH (horizontal dashed line) was used as a basis for the determination of ($\lambda_{\text{o}};\rho_{\text{o}}$). (**B**) Set of ($\lambda_{\text{o}};\rho_{\text{o}}$) couples that best reproduce the experimentally determined surface conductivity $K^{\sigma }$ at large pH. The contribution of electroosmosis to the total surface conductivity is given in terms of the ratio $K_{\text{eo}}^{\sigma }/(K_{m}^{\sigma }+K_{\text{eo}}^{\sigma })$ on the right axis. The ($\lambda_{\text{o}};\rho_{\text{o}}$) couple consistent with $K^{\sigma }$ and $I_{\text{str}}/\Delta P$ data is highlighted in blue. See the text and [26] for further details. Reprinted from [26]. Copyright 2011 with permission from Elsevier.

Figure 4A shows the dependence of the measured surface conductivity $K^{\sigma }$ on pH in 1 mM KCl solution concentration. $K^{\sigma }$ increases significantly with increasing pH above 4 and reaches a ~330 nS plateau value at pH ≥ 8. The surface conductivity and the layer thickness (not shown here) exhibit very similar dependence on solution pH, underlining that both quantities are inherently functions of the ionization level of carboxyl groups within the PAA film [26]. The increase in $K^{\sigma }$ with pH results from the accumulation of counterions within the film *and* at the interfacial EDL region outside the film, thereby compensating the structural film charge stemming from ionized carboxyl groups. Under action of an electric field, these ions migrate along the PE surface layer and thus contribute to $K^{\sigma }$. At sufficiently large pH (pH ≥ 8), dissociation of carboxyl groups is complete, which explains the presence of the plateau in $K^{\sigma }$ (Figure 2A).

A mean film thickness of ~18 nm was estimated from ellipsometry measurements at pH 2 [26]. For a given salt concentration, the film thickness increased with increasing pH and reached 60 to 70 nm at pH 10 [26]. In line with the stronger ionization of densely-packed carboxyl groups at higher salt concentrations (see the charge source term in Equation (1)), film swelling occurred in a lower pH range upon the increase of the KCl solution concentration [26]. As stated above, the increase in film thickness closely followed the increase in surface conductivity, meaning that surface conductivity mirrors the increasing counter ion concentration in the PAA film, a process that leads to increased osmotic pressure and enhanced film swelling.

With increasing charge density, electroosmosis may significantly contribute to the surface conductivity of the PE layer. $K^{\sigma }$ is then determined by the electrostatic properties of the film (contribution $K_{\text{m}}^{\sigma }$) and by its permeability to flow *via* the electroosmotic term $K_{\text{eo}}^{\sigma }$. Indeed, similar to the pressure-driven flow field V(X), the electroosmotic flow field $V_{\text{eo}}\left(X\right)$ depends on the quantity 1/*λ*_o_ (see Equation (3) and Section 2.5). Duval et al. developed a procedure (detailed below) to evaluate the migration ($K_{\text{m}}^{\sigma }$) and electroosmotic ($K_{\text{eo}}^{\sigma }$) components of the surface conductivity $K^{\sigma }$with consistent account of the measured variations of $I_{\text{str}}/\Delta P$ with changing pH and salt concentration [26].

The pronounced maximum observed for the streaming current at 0.1 mM KCl (Figure 3) was shown to originate from heterogeneous film swelling and the corresponding increase in the interphase diffuseness *α*, the latter length scale quantifying how far PAA polymer segments protrude from bulk film toward the outer electrolyte solution [26]. Indeed, as discussed in Section 3.1, an increase in *α* leads to enhanced friction exerted by the soft polymer interphase on flow, and thus, this results in decreased streaming current above a critical pH value, where swelling is most significant. Below that critical pH, the streaming current classically increases with pH as the result of the gradual dissociation of carboxylic groups. The maximum in the streaming current thus stems from interphase film swelling and the accompanied effects on developed hydrodynamic flow. Due to significant screening of film electrostatics, at fixed pH, the streaming current in a 10 mM KCl solution concentration is lower (in magnitude) compared to values measured in 1 and 0.1 mM KCl. More interestingly, the maximum disappears due to significant screening of film charges and to reduced Debye length compared to *α*, thus rendering impossible the electrokinetic probing of the segment density distribution at high salt concentrations. At pH ≥ 6, $I_{\text{str}}/\Delta P$ reaches a constant value, which suggests that ionization of the carboxyl groups is complete in this pH range.

Figure 4 shows that the increase in $K^{\sigma }$ with pH basically compounds that measured for $I_{\text{str}}/\Delta P$ (in magnitude) in 10 mM KCl solution. The latter result was exploited in Ref. [26] for deriving in a consistent way the relevant PAA electrohydrodynamic parameters $1/\lambda_{\text{o}}$ and $\rho_{\text{o}}/F$. Indeed, at pH ≥ 6, the magnitude of $K^{\sigma }$ is solely defined by these two quantities and hardly depends on *α*, which was verified *a posteriori*. The set of ($\lambda_{\text{o}}$;$\rho_{\text{o}}$) couples that best recover the plateau value reached by $K^{\sigma }$ at large pH is given in Figure 2B together with the corresponding ratio $K_{\text{eo}}^{\sigma }/{Κ}^{\sigma }$. The latter increases with increasing hydrodynamic penetration, expressed in terms of the Brinkman length $1/\lambda_{\text{o}}$ and the magnitude of the charge density required to reproduce $K^{\sigma }$ at large pH decreases with increasing $1/\lambda_{\text{o}}$. Considering the surface conductivity alone is therefore not sufficient for an unambiguous determination of the parameters $1/\lambda_{\text{o}}$ and $\rho_{\text{o}}/F$. Duval et al. [26] have shown that a consistent solution may be obtained by the additional evaluation of the dependence of $I_{\text{str}}/\Delta P$ on *α* in 10 mM KCl under complete ionization conditions (pH ≥ 6). This analysis was performed for the different couples ($\lambda_{\text{o}}$;$\rho_{\text{o}}$) that are in agreement with $K^{\sigma }$ data measured at large pH (as shown in Figure 2B). In line with theory on electrokinetics of diffuse soft thin films [25,26], $I_{\text{str}}/\Delta P$ decreases with increasing *α* and increases with increasing $1/\lambda_{\text{o}}$, as a result of the associated changes in the hydrodynamic flow-field (case discussed in Figure 2D at low to moderate $1/\lambda_{\text{o}}$). A comparison of the simulation results obtained for $I_{\text{str}}/\Delta P$ *vs. α* (Figure 1B) with the streaming current plateau value measured at large pH in 10 mM KCl solution (Figure 1A) finally provided the searched $\rho_{\text{o}}/F$ and $1/\lambda_{\text{o}}$ values, *i.e.*, $1/\lambda_{\text{o}}$ = 0.47 nm and $\rho_{\text{o}}/F$= 1635 mM [26]. The corresponding ratio $K_{\text{eo}}^{\sigma }/(K_{\text{m}}^{\sigma }+K_{\text{eo}}^{\sigma })$ is 0.32, confirming that electroosmotic charge transport contributes significantly to the overall surface conductivity $K^{\sigma }$ for polymer films with moderate to high charge density. Using $1/\lambda_{\text{o}}$ = 0.47 nm and $\rho_{\text{o}}/F$ = 1635 mM, the softness parameter *α* (and its modulation with pH and salt concentration) and the dissociation p*K* value pertaining to PAA carboxyl groups were subsequently obtained from reconstruction of the full dependence of $K^{\sigma }$ and $I_{\text{str}}/\Delta P$ on pH and salt concentration [26].

In summary, the developed strategy for the coupled analysis of the pH- and salt concentration-dependent streaming current, surface conductivity and swelling data allows for a comprehensive characterization of the charge and structure of polyelectrolyte layers. Strategies for polymer coatings without ionizable groups were further developed; see e.g., [24,28]. The gained information helps to better understand the fundamental properties and interactions of polymer systems, but it is also of interest for designing application-oriented materials, e.g., responsive or anti-fouling coatings.

### 3.3. Electrohydrodynamics of Polyelectrolyte Multilayers

Electrokinetic measurements are frequently applied to characterize the formation, charge and stability of polyelectrolyte multilayers (PEMs) [39,40,41,42]. Because of the inherent coupling between electrostatics and hydrodynamics in the development of soft surface electrokinetics, the unambiguous interpretation of data acquired on PEM materials requires concepts that extend the basic theory outlined in Section 2. Here, we review recent theoretical developments on electrokinetics of planar PEMs, and these developments are shown to reproduce, at least qualitatively, the peculiar electrokinetic properties of PEM that exhibit some degree of charge stratification absent from the theory outlined in Section 2.

The theory for the streaming current of PEMs was developed by Duval et al. for systems consisting of *N* alternately deposited cationic and anionic layers of thickness denoted as $\delta_{j}$, each being assigned an index j, with j=1,...,N, where j=1 stands for the most internal layer directly supported by the substrate surface and j=N for the outermost layer at the PEM/electrolyte solution interface [27]. Each layer *j* is considered to carry a single type of ionisable groups with a given p*K* and ε value (denoted in [27] as p*K_j_* and *ε_j_*, see analogy to Equation (1)). Furthermore, the theory is applicable for any spatial distribution of polymer segments and structural charge density from the hard supporting surface to the outer solution medium without limitations on thickness and charge magnitude pertaining to each layer constituting the PEM. The theoretical framework [27] further ensures a smooth transition of the segment density between adjacent layers, each being attributed a given hydrodynamic softness $\lambda_{j}$ and charge density $\rho_{j}/F$, the analogue of the quantities $\lambda_{\text{o}}$ and $\rho_{\text{o}}/F$ introduced in Section 2 for monolayered films.

The electrostatic potential distribution across PEMs may be obtained by solving the non-linear Poisson–Boltzmann equation that includes terms for the structural charges resulting from the ionizable groups in each sub-layer and for mobile counter ions distributed inside and outside the PEM [27]. The hydrodynamic flow field can be calculated on the basis of the Brinkman Equation (3) taking into account the impact of the local density of polymer segments on the friction they exert on flow under electrokinetic conditions. The expressions given in Section 2 for the calculation of the streaming current and surface conductivity remain valid for PEMs.

In analogy to monolayered systems, the magnitude and sign of the streaming current of PEMs is determined by the spatial extension of the electrokinetically-active film region, *i.e.*, the zone where film electrostatic properties are probed by the pressure-driven flow penetrating the film at a finite thickness. The extension of that electrokinetically-active outer film region depends not only on the hydrodynamic film permeability determined by the polymer segment density distribution, but also on the Debye layer thickness fixed by solution ionic strength. Streaming current measurements performed at PEMs may therefore reflect electrohydrodynamic contributions from internal layers embedded within PEMs and not only those from layers that directly face the outer electrolyte solution. As a consequence, the comprehensive electrokinetic characterization of PEMs requires measurements at varying pH values and different salt concentrations of a neutral background electrolyte in order to tune the Debye layer thickness and therewith the outer film region that is effectively probed by electrokinetic flow. Applying this strategy, the contributions of the different layers constituting the PEM may be evaluated, and the differentiated impacts of, e.g., outer polyanionic and inner polycationic layers are reflected by a salt concentration-dependent shift in the point of zero streaming current (PZSC), defined by the pH value where $I_{\text{str}}/\Delta P=0$. This remarkable PEM electrokinetic feature is now discussed in some more detail.

**Figure 5:**
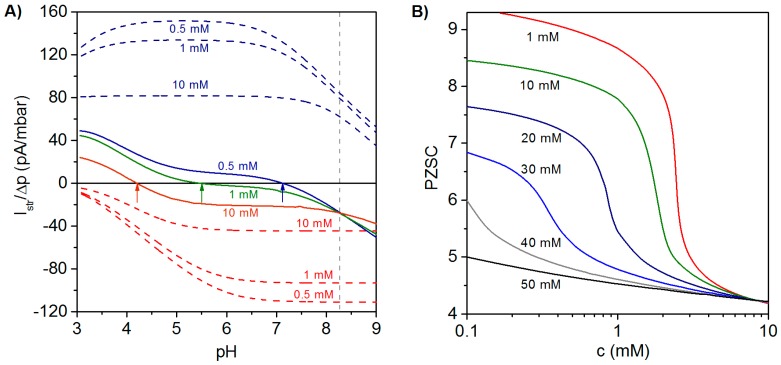
(**A**) Ratio of streaming current over applied pressure ($I_{\text{str}}/\Delta P$ ) *vs.* pH evaluated at three electrolyte concentrations (indicated) for a polycationic layer (blue dashed lines), a polyanionic layer (red dashed lines) and a polycation (j=1 )/polyanion (j=2 ) bilayer film (colored solid lines). Model parameters for the bilayer system: $\text{p}K_{1}=9$, $\varepsilon_{1}=+1$, $\delta_{1}=30$ nm, $\text{p}K_{2}=4$, $\varepsilon_{2}=-1$, $\delta_{2}=5$ nm, $\left|\rho_{1,2}/F\right|=20$ mM, $1/\lambda_{1,2}=4.5$ nm; in each layer, a homogeneous distribution of polymer segments is considered. Model parameters for the polycation layer and polyanion layer are similar to those adopted for the polycationic and polyanionic layers forming the bilayer film. Arrows highlight the decrease of point of zero streaming current (PZSC) for the bilayer film with increasing electrolyte concentration. (**B**) PZSC *vs.* electrolyte concentration for a bilayer system consisting of a polycationic layer with fixed charge density and a polyanionic layer with varying charge density (indicated in terms of equivalent concentration of charges). Other parameters as in the left panel. Adapted from [27]. Copyright 2011 with permission from American Chemical Society.

Illustrative simulation results for the dependence of $I_{\text{str}}/\Delta P$ on pH and electrolyte concentration are shown in Figure 5A for a polyelectrolyte bilayer film consisting of a polycationic layer (thickness $\delta_{1}=30\;\mathrm{nm}$, $\text{p}K_{1}=9$, $\varepsilon_{1}=1$) immobilized on a hard and uncharged carrier, and a polyanionic layer (thickness $\delta_{2}=5\;\mathrm{nm}$, $\text{p}K_{2}=4$, $\varepsilon_{2}=-1$) located at the solution side of the bilayer construction [27]. For the sake of comparison, the variation of $I_{\text{str}}/\Delta P$ with pH and electrolyte concentration is shown for single polycationic and polyanionic layers. For the monolayered polycationic and polyanionic systems, the position of the PZSC and the dependence of $I_{\text{str}}/\Delta P$ on solution pH are conform to the intrinsic ionization characteristics of the polyelectrolytes and to the compression of the electric double layer with increasing solution ionic strength that leads to reduction of $\left|I_{\text{str}}/\Delta P\right|$ at given pH [26,27].

The electrokinetic fingerprint of the polyelectrolyte bilayer shows two remarkable features: (i) For a given electrolyte concentration, $I_{\text{str}}/\Delta P$ changes its sign from negative values at high pH to positive values at low pH. The PZSC (*i.e.*, the pH where sign reversal occurs) is shifted to higher values with decreasing salt concentration. These results thus reveal an increasing contribution of the inner cationic layer to the streaming current at sufficiently low ionic strengths. In addition, Figure 5A highlights the necessity to vary pH and salt concentration over broad ranges of values in order to achieve an unambiguous electrokinetic characterization of PEMs. (ii) The curves $I_{str}/\Delta P$ *vs*. pH obtained at different salt concentrations exhibit a common intersection point at pH ~8.27. The occurrence of this feature is expected for multilayered systems provided that the thickness of the constituting layers is independent of the concentration of the background electrolyte and of pH. Changes in the layer structure would lead to varying contributions of the various layers to $I_{\text{str}}/\Delta P$ and, as a consequence, to the absence of common intersection point. Figure 5B illustrates the dependence of the PZSC on salt concentration for different densities of charges $\left|\rho_{2}\right|$ in the topmost (anionic) layer, while all other model parameter values are identical to those adopted in Figure 5A. At a fixed salt concentration, the origin of the change in PZSC towards acidic pH values with increasing the charge density (in magnitude) of the outermost anionic layer is the decrease of the electrical potential in the electrokinetically-active film region. Under such conditions, the potential in that zone becomes significantly affected by the electrostatic features of the external layer, which leads to a decrease in PZSC with increasing $\left|\rho_{2}\right|$. At a fixed charge density of the anionic layer, increasing salt concentration leads to an electric double layer that becomes mostly confined within the outer layer. The latter then predominantly governs the electrokinetic properties of the PEM as a whole, which results in the observed decrease of the PZSC with increasing salt concentration. This decrease is most pronounced for cases where the magnitudes of charge densities in cationic and anionic layers are significantly different from each other, which exacerbates their differentiated respective contributions upon increasing solution ionic strength. A detailed analysis given in [27] further demonstrates that the PZSC of PEMs is strongly determined by the dissymmetry in p*K* values of the ionogenic groups in each constituting layer, by the respective thickness of the layers and by their segment density distribution. Furthermore, the relevance of the theoretical framework outlined in this section for PEMs (in particular, the unconventional dependence of their PZSC on salt concentration) was confirmed by experimental results obtained on a polyelectrolyte bilayer consisting of PEI (poly(ethylene imine)) and PAA and on an anionic lipid bilayer supported by a polycationic PEI cushion [27].

In summary, the PZSC of PEMs is determined by the complex interplay between pH- and ionic strength-dependent electrohydrodynamic processes that govern the charging, charge compensation and structural properties of the various layers constituting the PEM. For this reason, the PZSC measured for soft multilayered films does not correspond to the pH value where the overall film charge is zero. As a result, the comprehensive electrokinetic characterization of PEMs requires systematic measurements performed as a function of pH and salt concentration. Finally, it is stressed that the electrohydrodynamics and the point of zero mobility of charge-stratified particles exhibit similar characteristics as those discussed here for planar systems. A comprehensive overview about theory, experiments and simulations on charge-stratified particulate systems can be found in [43,44,45,46]. As an illustrative example, the point of zero electrophoretic mobility for poly(amidoamine) starburst dendrimers with a positive internal core and a negatively-charged outer shell layer strongly decreases with increasing salt concentration for the same reasons invoked here for planar charge-stratified polyelectrolyte systems.

## 4. Conclusions and Perspectives

The electrohydrodynamics of soft surfaces is strongly determined by the distribution of charged polymer segments across the film/electrolyte interface. Electrokinetics therefore provides valuable options for analyzing the interfacial charge and structure of soft surfaces in contact with aqueous solutions. Here, we report and discuss the theory and strategies for the analysis of the interfacial charge and structure of planar polyelectrolyte layers from the measurement of streaming current, surface conductivity and film swelling properties. General trends are illustrated and discussed on the basis of simulation results. As specific examples, we report data of the analysis of charge and structure of PAA thin films and discuss remarkable electrokinetic features of charge-stratified interphases on the basis of simulation results. Overall, electrokinetics is shown to be a spatially-resolved technique suitable for deciphering the interfacial charge and structure of soft polymeric coatings within a so-called electrokinetically-active outer film region whose extension is fixed by the electric double layer thickness and by the film permeability. Future developments will have to aim at a better understanding of the interactions between multivalent ions or charged molecules and polyelectrolyte layers. In combination with other methods, surface conductivity measurements can be expected to provide additional information about the amount of ionized groups involved in the formation of salt bridges between oppositely-charged layers and to allow the analysis of changes in layer structure upon variations of adjacent electrolyte composition. The authors also anticipate that electrokinetic analysis of electroactive polymer films in the presence of redox active analytes in solutions could be decisive to investigate electron-transfer reactions at modified soft electrodes under lateral flow conditions. Depending on (bipolar) electrode kinetic properties, such systems are indeed expected to give rise to the non-linear dependence of streaming current on applied pressure. While analyses of this type have been conducted on bare (uncoated) conducting materials with resulting applications on the detection of redox active analytes, basic investigations of electron-conducting soft polymer electrokinetics are to the best of our knowledge still lacking in the literature. Finally, tackling the electrokinetics of soft planar polyelectrolytes at a molecular level using theories that go beyond the mean-field Poisson–Boltzmann level could pave the way for apprehending ion specificity effects, as well as the impacts of film structure on ions/particles selectivity or flow permeability.
